# Hypothermic in the Heat: A Case of Hypothermia in a Vulnerable Older Adult in South Florida

**DOI:** 10.7759/cureus.59091

**Published:** 2024-04-26

**Authors:** Katherine J Salim, Ronald Garry

**Affiliations:** 1 Internal Medicine, Naples Comprehensive Health (NCH) Healthcare System, Naples, USA; 2 Geriatrics, Naples Comprehensive Health (NCH) Healthcare System, Naples, USA

**Keywords:** hypothermia, geriatrics, core body temperature, active external rewarming, homeostasis

## Abstract

Hypothermia is defined as a core body temperature of less than 35°C. This report centers on the case of an older adult who presented to the emergency department (ED) with mild hypothermia, bradycardia, and electrolyte abnormalities during the summer in a warm climate. The patient was an 82-year-old man who was found to be hypothermic (33.6°C rectally), hypotensive, and bradycardic. He was treated with intravenous (IV) fluid resuscitation, active external rewarming (AER), and empiric antibiotics for his left lower lobe pneumonia. He was admitted to the intensive care unit and ultimately discharged home with physical therapy. Older adults are at an increased risk for hypothermia, even in a tropical climate. Early recognition of hypothermia is essential to achieve good outcomes.

## Introduction

Hypothermia is defined as a core body temperature of less than 35°C. It can be further classified as mild (32‐35°C), moderate (28‐32°C), and severe hypothermia (<28°C) [1]. There are approximately 1300 deaths per year in the United States due to hypothermia [2], and it is most commonly seen during winter months in the Northeast and the Midwest [3]. 

Older adults are more susceptible to hypothermia because of age‐related impairment in temperature homeostasis [4]. This impairment is partly due to a loss of elasticity in blood vessels that limits vasoconstriction and dilation, which in turn undermine temperature regulation via the skin. Older adults also have sarcopenia, which decreases their metabolic rate and ability to shiver [5]. Hypothermia has been found to be associated with extreme age, severe illness, medications, living alone, and cold weather [6]. On initial presentation to the emergency department, patients diagnosed with sepsis and hypothermia have higher mortality rates; thus, early recognition and aggressive treatment are imperative [7]. 

The current case highlights an older adult who presented with mild hypothermia, bradycardia, and electrolyte abnormalities that were precipitated by sepsis. The case presented here occurred during the summer in a warm climate, where hypothermia would not typically be expected. We review the risk factors, management, and prevention of hypothermia in elderly patients.

## Case presentation

An 82-year-old man presented to the emergency department (ED) in Naples, Florida, via nonemergency medical service owing to abdominal pain. He had presented six months previously with upper gastrointestinal bleeding from a duodenal ulcer. Three days prior to admission, he developed nausea, vomiting, anorexia, and epigastric pain. The patient was obese (31.6 kg/m2) and had a history of subclinical hypothyroidism and malnutrition. He had no recent falls and did not consume alcohol. The patient’s daily home medications included 40 mg of pantoprazole, 324 mg of ferrous gluconate, and 500 mg of vitamin C.

In the ED, he was mildly hypothermic, bradycardic, and hypotensive with a systolic blood pressure of 65 mmHg and a mean arterial pressure of 49 mmHg. On physical examination, he appeared chronically ill and was somnolent but easily arousable. He had mild epigastric tenderness to palpation. 

Laboratory tests were significant for glucose (66 mg/dL), sodium (125 mmol/L), magnesium (1.6 mg/dL), lipase (439 U/L), procalcitonin (0.12 ng/mL), thyroid-stimulating hormone (8.07 mlU/L), thyroxine (1.27 ng/dL), albumin (2.8 g/dL), total protein (7.4 g/dL), hemoglobin (11.4 g/dL), white blood cell count (4.3 x 10^3^/μL), relative neutrophils (66.3%), relative lymphocytes (22%), relative monocytes (9.3%), relative eosinophils (1.2%), relative basophils (0.2%), blood urea nitrogen (13 mg/dL), and creatinine (0.6 mg/dL). 

**Figure 1:**
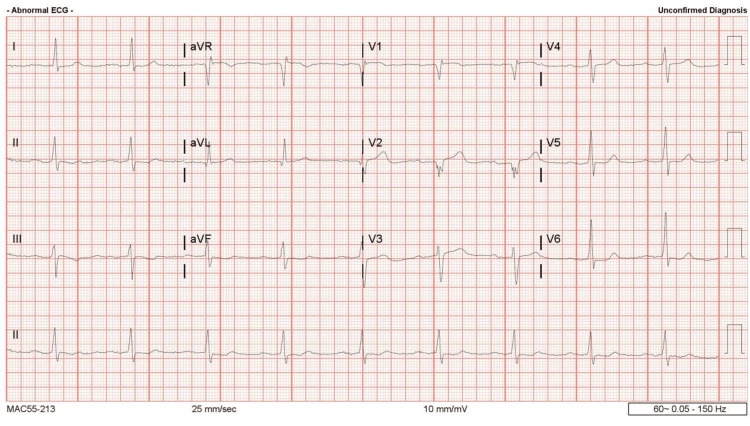
Electrocardiogram revealing sinus bradycardia with a first-degree atrioventricular block, with a heart rate of 56 beats per minute.

**Figure 2:**
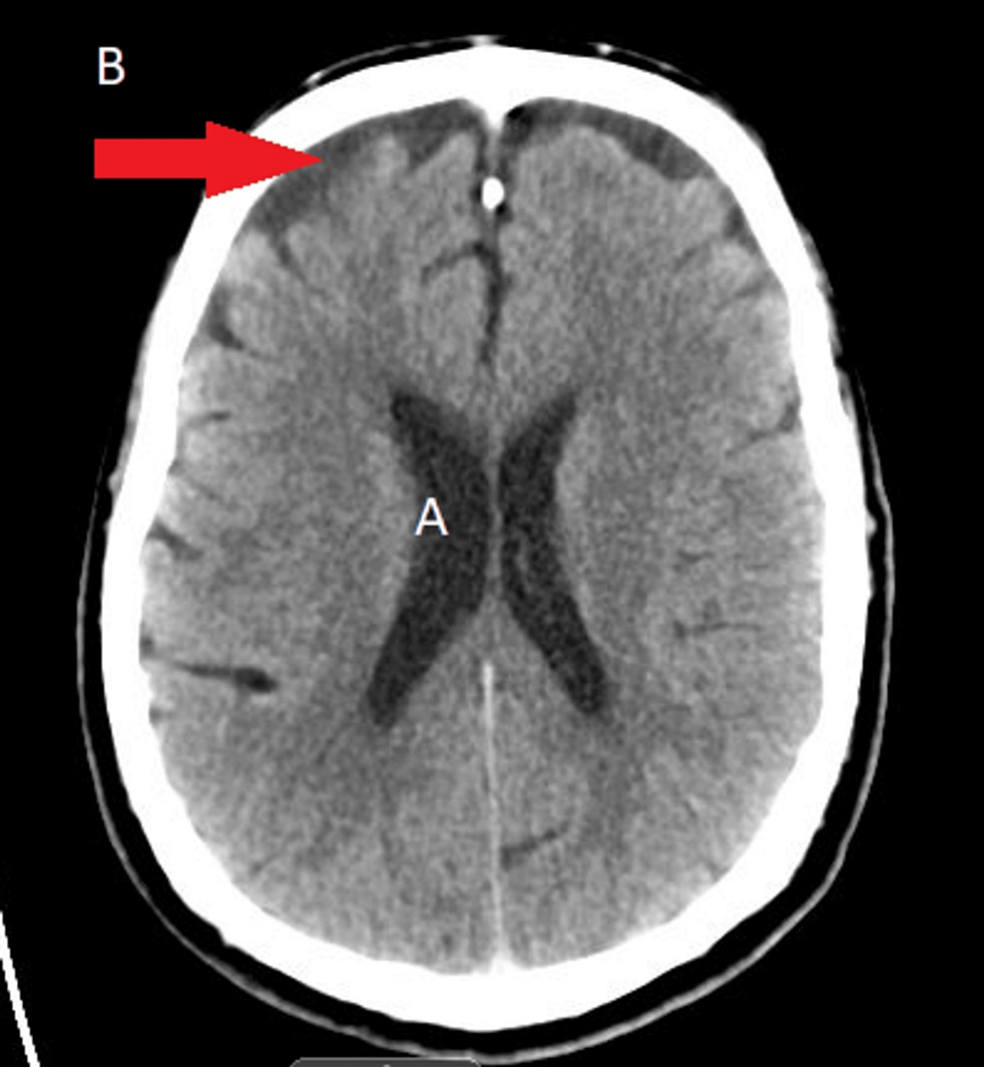
Computed tomography of the head without intravenous contrast. Ventricles demonstrated no evidence of hydrocephalus (A), but mild cortical atrophy was observed (B).

**Figure 3:**
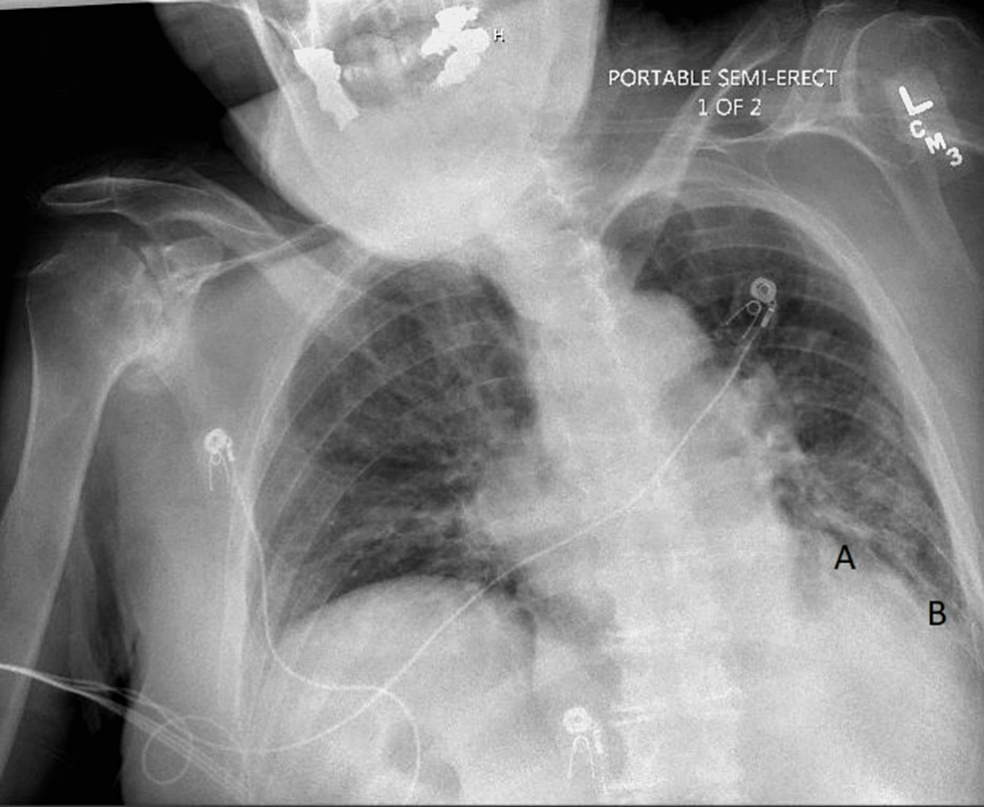
Portable 1 view chest X-ray with poor inspiratory effort. Overlay area of consolidation total suggestive of left lower lobe pneumonia (A), with blunted left diaphragm due to effusion with limited visualization due to rotation (B).

**Figure 4:**
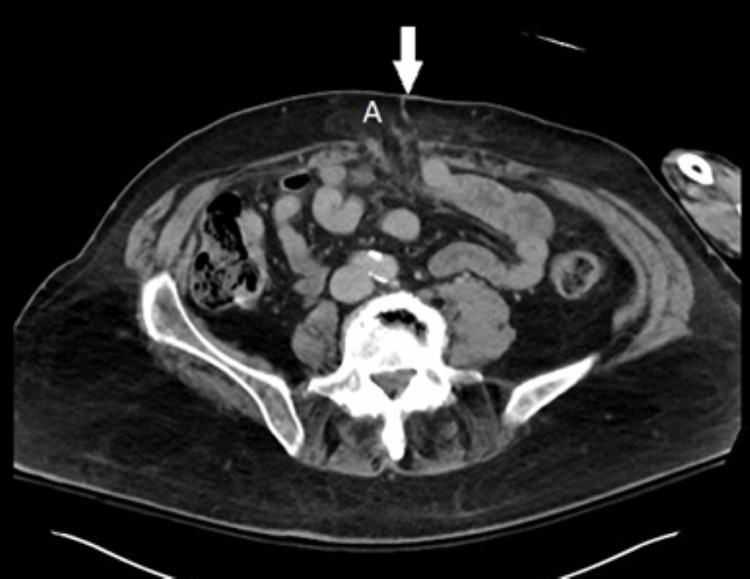
Computed tomography of the abdomen and pelvis, cross‐sectional view demonstrating a 5 cm midline suprapubic anterior abdominal wall hernia containing fat (A), with the left hand artifact.

**Figure 5:**
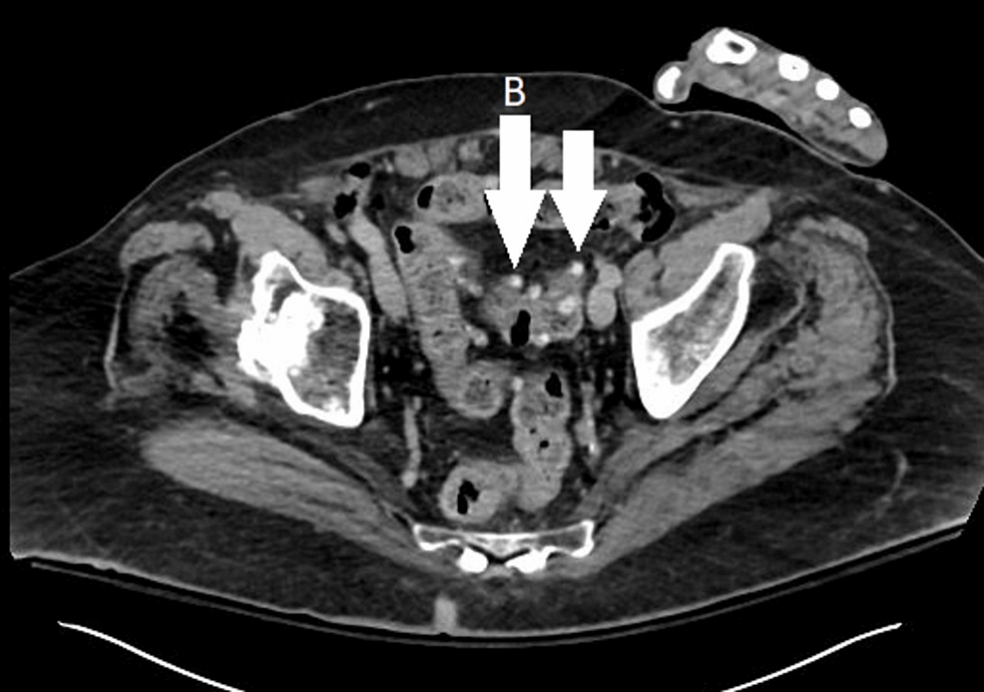
Computed tomography of the abdomen and pelvis, cross‐sectional view showing diverticulosis of the colon (most prominent in the sigmoid), but no evidence of diverticulitis (B) with the left hand artifact.

An electrocardiogram (EKG) revealed sinus bradycardia with a known first-degree atrioventricular (AV) block (present for years) and no evidence of a J wave (Figure 1). Computed tomography (CT) of the head was completed to exclude a central nervous system source of his hypothermia, and the scan was negative for acute abnormality (Figure 2). X‐ray imaging of the chest showed a left lower lobe consolidation and mild cardiomegaly (Figure 3). Abdominal CT imaging revealed a midline suprapubic abdominal wall hernia containing fat (Figure 4) and the presence of known sigmoid diverticulosis (Figure 5).

In the ED, the patient was resuscitated with intravenous (IV) fluids, active external rewarming (AER), empiric antibiotics (2 g of ceftriaxone and 250 mg of azithromycin), and 100 mg of hydrocortisone intravenously for the possibility of adrenal insufficiency. His temperature improved to 36°C with AER within three hours of presentation, and he had no further issues of hypothermia during his hospital stay. During his stay, his adrenocorticotropic hormone and morning cortisol levels were normal, and steroids were discontinued. The patient’s hypovolemic hyponatremia improved with IV fluids, and his magnesium was repleted. He was treated for sepsis related to his left lower lobe pneumonia, but blood culture data were negative, and no causative organism was identified. He finished a short course of antibiotics, and he was ultimately discharged home with physical therapy.

## Discussion

Hypothermia is an example of the atypical presentation of disease in older adults compared with younger adults, who commonly present febrile. Thermal regulation of the body is achieved in three ways: production of heat by muscles, heat exchange between the body and environment, and evaporation of sweat [5]. Normal aging can result in failures to conserve heat due to decreased vasoconstriction and a reduced ability to generate heat due to sarcopenia. Decreases in core body temperature of 10% can be life-threatening [5]. 

Homeostenosis is a core principle of geriatrics because older adults have a reduced ability to maintain homeostasis. It usually starts in the third decade of life, and rates of decline can be affected by disease states, environmental factors, and genetics [8]. An impaired ability to maintain the core body temperature is compounded by chronic disease states such as hypertension that are often treated with medications that can themselves impair homeostasis [5]. Medications that can significantly affect thermoregulation include beta blockers, which impair heat production; calcium channel blockers and alpha blockers that decrease vasoconstriction; and hypoglycemics that deplete substrates required for thermogenesis [5,9]. 

Our patient’s glucose was 66 mg/dL, likely related to malnutrition and decreased glycogen stores from rapid weight loss. This low glucose level likely contributed to our patient’s diminished ability to shiver and generate heat. Shivering also uses glycogen stores, which likely further contributed to his hypoglycemia. During rewarming, moderate degrees of hyperglycemia are acceptable [1,10].

The patient also presented with sinus bradycardia and first-degree AV block on EKG. He was not taking beta blockers prior to presentation. Bradycardia is seen with hypothermia due to a decreased spontaneous depolarization of the pacemaker cells [1] and a prolonged contraction-relaxation cycle [11]. Other significant EKG findings in hypothermia include J waves, QT prolongation, and QRS widening [12,13]. In the current case, the patient was monitored on telemetry owing to the risk of ventricular arrhythmias. 

Treatment of hypothermia involves generalized supportive measures. It is imperative to resuscitate within six hours of presentation because hypothermia is associated with increased mortality [14]. Rewarming techniques include passive external rewarming (PER), AER, and active core rewarming (ACR). PER involves removing wet clothing and providing warm blankets to support a patient’s own heat production. PER is often used in conjunction with AER. AER consists of providing external heat via a heating blanket or a tub of warm water [5]. ACR involves direct heating of the core via warmed IV fluids, peritoneal dialysis, bladder irrigation, or extracorporeal methods, and it is generally reserved for severe hypothermia [5]. In our patient, AER was completed, with body temperature rising appropriately.

Our case has broader implications for warm temperature climates. General measures that limit sarcopenia such as weight training may attenuate risk factors for hypothermia. A Mediterranean diet may improve vascular health, which therefore may improve thermoregulation [15]. It is also important to educate patients on the importance of having an adequate fluid and calorie intake and avoiding excessive alcohol consumption [16], even during summer months.

## Conclusions

Due to impaired thermoregulatory ability, older adults, even in a tropical climate, are more susceptible to hypothermia. Hypothermia is an atypical presentation of infection, and it can be easily missed in the elderly since they may appear warm peripherally due to their impaired ability to vasoconstrict. Early recognition of hypothermia is imperative for improved clinical outcomes, especially in the elderly population. If hypothermia is suspected, a core body temperature should be taken, which is usually done rectally.
